# An outlook to sophisticated technologies and novel developments for metabolic regulation in the *Saccharomyces cerevisiae* expression system

**DOI:** 10.3389/fbioe.2023.1249841

**Published:** 2023-10-05

**Authors:** Yijian Wu, Sai Feng, Zeao Sun, Yan Hu, Xiao Jia, Bin Zeng

**Affiliations:** College of Pharmacy, Shenzhen Technology University, Shenzhen, Guangdong, China

**Keywords:** metabolic regulation, promoter engineering, fusion partner, genomic engineering, CRISPR/Cas9, codon optimization

## Abstract

*Saccharomyces cerevisiae* is one of the most extensively used biosynthetic systems for the production of diverse bioproducts, especially biotherapeutics and recombinant proteins. Because the expression and insertion of foreign genes are always impaired by the endogenous factors of *Saccharomyces cerevisiae* and nonproductive procedures, various technologies have been developed to enhance the strength and efficiency of transcription and facilitate gene editing procedures. Thus, the limitations that block heterologous protein secretion have been overcome. Highly efficient promoters responsible for the initiation of transcription and the accurate regulation of expression have been developed that can be precisely regulated with synthetic promoters and double promoter expression systems. Appropriate codon optimization and harmonization for adaption to the genomic codon abundance of *S. cerevisiae* are expected to further improve the transcription and translation efficiency. Efficient and accurate translocation can be achieved by fusing a specifically designed signal peptide to an upstream foreign gene to facilitate the secretion of newly synthesized proteins. In addition to the widely applied promoter engineering technology and the clear mechanism of the endoplasmic reticulum secretory pathway, the innovative genome editing technique CRISPR/Cas (clustered regularly interspaced short palindromic repeats/CRISPR-associated system) and its derivative tools allow for more precise and efficient gene disruption, site-directed mutation, and foreign gene insertion. This review focuses on sophisticated engineering techniques and emerging genetic technologies developed for the accurate metabolic regulation of the *S. cerevisiae* expression system.

## 1 Introduction

Since its domestication, the unicellular eukaryote *Saccharomyces cerevisiae*, also known as budding yeast, has become one of the most extensively used microorganisms for heterologous protein production (Borodina and Nielsen, 2014; den Haan et al., 2021). Molecular biology and genetic manipulation techniques have already been devised and applied; both the whole genome sequence (Goffeau et al., 1996) and sufficient omics data of *S. cerevisiae* are also accessible. Specifically, as outlined by the Food and Drug Administration, *S. cerevisiae* is generally regarded as safe for the production of food and biopharmaceuticals. Moreover, because of its clear genetic background, simple manipulation, rapid growth rate, posttranslational processing ability, and capability to secrete heterologous proteins in their native forms (Buckholz and Gleeson, 1991; Parapouli et al., 2020), *S. cerevisiae* has been considered a “eukaryotic *Escherichia coli*” and “*in vivo* test tube”. *Saccharomyces cerevisiae* has become an ideal cell factory for the biosynthesis of heterologous proteins and metabolites without any detectable endotoxins that are pathogenic to humans (Borodina and Nielsen, 2014; Cho et al., 2022).

Because of its excellent posttranslational modification ability that is analogous to that of higher eukaryotes and allows for simplifying downstream protein recovery and purification processes (Calado et al., 2004), *S. cerevisiae* is used for the production of numerous complex proteins applied in biopharmaceuticals and the bioindustry. The expression procedures of heterologous proteins by *S. cerevisiae* generally comprise the construction of an expression vector in *E. coli* carrying the gene of interest (GOI) and the introduction of the constructed expression vector into *S. cerevisiae* (Gonzalez et al., 2019). Although most posttranslational modifications that occur in higher eukaryotic cells can be accomplished by *S. cerevisiae*, heterologous protein production always remains at a low level (Ilmen et al., 2011).

Overall, while *S. cerevisiae* is a powerful expression system that offers many advantages, it is important to carefully consider the following limitations associated with the procedures to synthesize bioproduct varieties: i) its inability to perform certain eukaryotic post-translational modifications such as glycosylation, phosphorylation, and sulfation affect the biological activity and stability of bioproducts to a certain extent, particularly those requiring specific modifications to function properly; ii) the relatively low secretion capacity of *S. cerevisiae* limits the production of soluble and functional proteins that require secretion for proper folding and stability; iii) preferred genetic codon usage leads to suboptimal protein expression from heterologous genes with different codon usage biases. Recent advances in synthetic biology and genetic engineering have substantially helped to overcome these bottlenecks of *S. cerevisiae* heterologous expression systems. Promoters are responsible for regulating the initiation of foreign gene transcription and the optimization of metabolic pathways, which directly impact target gene expression. The developed double promoter expression systems can be expected to notably improve the transcription level of target genes. A stable and high-level transcription strength always requires the intensive folding capability of the endoplasmic reticulum (ER), which commonly results in the activation of unfolded protein response (UPR) to buffer the high folding demand (Sharma et al., 2022). The extra upregulation of the expression level of folding factors and functional chaperones that reside in the ER lumen enables an elevation of the ER folding capability (Young and Robinson, 2014). To further facilitate the post-translation and secretion process, fusion partners present as oriented functionalities that are commonly fused to target units for efficient translocation, accurate co-localization, and high production enhancement (Bae et al., 2015). This has emerged as the preferred option. Moreover, the innovative genome editing technique of CRISPR/Cas (clustered regularly interspaced short palindromic repeats/CRISPR-associated system) and its derivative tools enable more precise and efficient gene manipulation. Appropriate codon optimization and harmonization for adaption to the genomic codon usage bias of *S. cerevisiae* are expected to increase both the biosynthesis efficiency and bioproduction level. Hence, this review focuses on both sophisticated and innovative technologies that have been developed to overcome the bottlenecks limiting the production of heterologous and recombinant proteins (r-proteins) as well as to meet the increasingly growing demand for therapeutics and pharmaceutics. Examples include promoter engineering for powerful transcription, fusion partners for efficient translocation and high-level secretion, and modification of the ER secretory pathway in the case of undesired degradation; further examples are precise genome engineering for target insertion, mutation, and disruption, metabolic engineering for newly constructed functional hosts, as well as other emerging and promising techniques for the simplification of expression procedures. The combination of conventional gene manipulation strategies and novel nucleotide-level editing techniques always achieves better outcomes.

## 2 Well-developed technologies used in *Saccharomyces cerevisiae* expression systems

### 2.1 Promoter engineering in the metabolic pathway

For the expression of a foreign target protein, a promoter, a secretion signal sequence, the target protein sequence, and a terminator are indispensable elements during the whole secretory process. To initiate foreign gene transcription, the selection of a powerful promoter is one of the most important factors affecting the efficiency and intensity of transcription (Struhl, 1995; Redden et al., 2015). Appropriate selection and specific modifications of promoters usually lead to more efficient secretion and higher protein production (Baldari et al., 1987; Ruohonen et al., 1995; Li et al., 2019). As heterologous promoters generally display a poor expression level, homologous promoters originating from yeasts have become the preferred option (Mattanovich et al., 2012). Promoter modification can be accomplished with random strategies, including site-directed mutagenesis, sequence randomization, error-prone PCR, and hybrid promoter engineering (Xu et al., 2019). Although increasingly developed molecular biology and biosynthetic platforms, as well as the combination of synthetic biology with high-throughput tools are expected to facilitate and establish revolutionary promoter expression systems (Vaishnav et al., 2022), the effect of artificial regulation on metabolic pathways related to the transcription of yeast promoters remains complicated and unpredictable (Peng et al., 2022a).

#### 2.1.1 Inducible promoters

Inducible promoters are activated to initiate gene transcription or repressed to decrease target expression by the addition of stimuli to the medium. They have been developed to be applied for the optimization of heterologous metabolic pathways and regulatory networks for accurate gene mediation. Glycolytic promoters, the most powerful promoters of *S. cerevisiae* obtained from genes encoding glycolytic enzymes, are the first promoters applied in protein production, and can be induced by glucose addition to the medium (Rajkumar et al., 2019). In contrast, galactose-regulated promoters, which are strongly repressed by glucose and generated from genes involved in metabolizing galactose (such as *GAL1*, *GAL2*, *GAL7*, and *GAL10*) (Romanos et al., 2001; Peng et al., 2017), are the most powerful and tightly regulated promoters in *S. cerevisiae*. Those GAL promoters have been used in metabolic engineering approaches for the reconstruction of complicated heterologous metabolic pathways and in genetic engineering for stable genetic manipulations. The glucose-repressible promoters ADH2 (alcohol dehydrogenase 2) (Peng et al., 2015), invertase gene *SUC1* (Rodriguez et al., 1981), and invertase gene SUC2 are strongly repressed by the addition of glucose while they are activated by reducing glucose concentration in the medium (Sharma et al., 2022). The powerful and tightly-regulated promoter ADH2 has been used for the expression of heterologous genes (especially genes encoding toxic proteins, e.g., insulin-like growth factor I) owing to its trait of being heavily repressed by glucose (Shuster, 1989; Price et al., 1990). Promoters that are not induced by the nutrients in the medium can also be used for heterologous gene expression; these include temperature-regulated promoters, steroid-regulated promoters, foreign promoters, and the copper-induced promoter CUP1. CUP1 is responsible for moderate transcriptional strength under a certain copper concentration, which is typically used to avoid metabolic imbalance during the log growth phase or post-log phase without extra copper addition. Although copper-induced expression systems show a high copy number and stable transcriptional strength, the repeatedly used *CUP1* gene tends to result in a loss of introduced GOI by homologous recombination. Synthetic regulatory modules for gene transcription are expected to reduce genetic instability caused by a reduplicated induction system for metabolic modification and regulatory introduction of heterologously metabolic pathways (Peng et al., 2017).

#### 2.1.2 Constitutive promoters

While inducible promoters have the capability to maintain the yeast culture at a non-expression status during the cell growth phase, which results in the minimization of the selection for non-expression mutants (Mattanovich et al., 2012), constitutive promoters seem to be preferred. Compared to regulated inducible promoters, constitutive promoters are unregulated, which means that the transcription efficiency of a heterologous gene controlled by constitutive promoters is almost unaffected by both internal and external factors. Moreover, inducible promoters are generally activated under specific circumstances (e.g., addition of biotic or abiotic stimuli), and commonly negatively affect the purity and concentration of target proteins, even requiring a more rigorous isolation strategy. Notably, constitutive promoters are responsible for the application of the expression of both intracellular and extracellular proteins by *S. cerevisiae*, yet overexpression of a target protein often specifically depends on the target protein being expressed in *S. cerevisiae* (Da Silva and Srikrishnan, 2012). For example, the promoter ADH1, which is one of the most extensively utilized constitutive promoters for the production of heterologous proteins in yeast, naturally controls the expression of alcohol dehydrogenase 1 in *S. cerevisiae*. Another constitutive promoter (pTEF1) controls the expression of the translation elongation factor EF1 alpha, showing higher expression intensity than promoter alcohol dehydrogenase 1 and being unrepressed by ethanol (Partow et al., 2010). pTEF1 has been used to enhance the production of r-proteins and homologous enzymes by *S. cerevisiae* (Steinborn et al., 2007; Zhang et al., 2017; Xiao et al., 2023). Other constitutive promoters have also been applied to the construction of expression vectors (Partow et al., 2010; Lee et al., 2013). For example, Pgpd (which encodes glyceraldehyde 3-phosphate dehydrogenase) is used to regulate heterologous or recombinant gene expression processes (Knudsen et al., 2015); pPGK1 (which encodes phosphoglycerate kinase 1) is used for accurate metabolic and transcriptional regulation (Lamour et al., 2019; Jin et al., 2020; Shi et al., 2022); pTPI1 (which encodes triosephosphate isomerase) is used to precisely regulate protein expression and markedly increase industrial product production by *S. cerevisiae* (Chen et al., 2019; Azizoglu et al., 2021; Olzhausen et al., 2021; Paramasivan et al., 2022); pHXT7 (which encodes a hexose transporter) is used to improve the sugar transportation efficiency in the metabolic pathways of *S. cerevisiae* (Vasylyshyn et al., 2020; Zhu et al., 2021); and pPYK1 (which encodes pyruvate kinase 1) is used to rewire certain metabolic networks (Kim et al., 2016; Zhang et al., 2019). Although constitutive promoters are the most used promoters for foreign gene expression because of their unimpaired transcriptional strength, the effects of various culture conditions on heterologous metabolic pathways are unpredictable.

#### 2.1.3 Promising double-promoter expression systems

**FIGURE 1:**
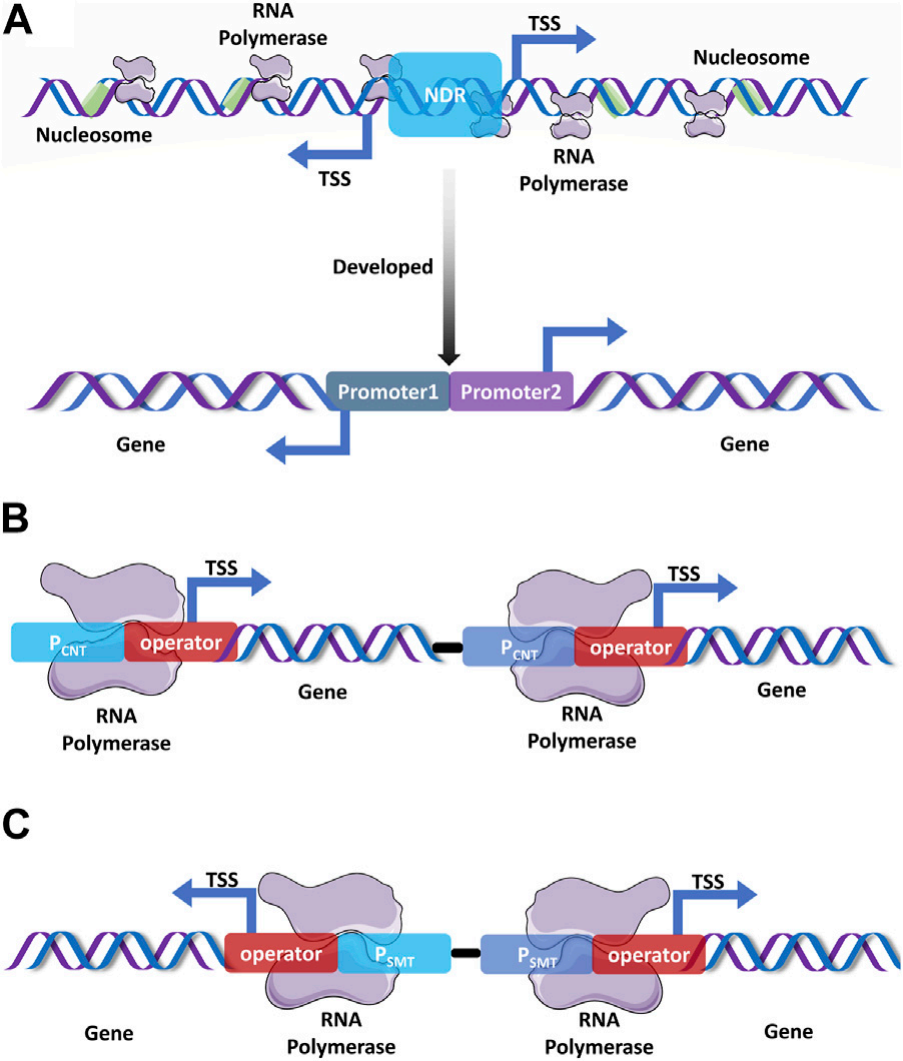
Promising promoter expression systems. **(A)**. The bidirectional promoter that is generated from yeast initiates transcription in both orientations as transcription start sites (TSSs) are located in the same nucleosome-depleted region (NDR) (Xu et al., 2009; Wei et al., 2011), which is responsible for the assembly and origination of gene transcription. Construction of a double promoter expression system by the fusion of two powerful promoters based on the bidirectional promoters from *S. cerevisiae* (Miller et al., 1998; Yuan et al., 2021). **(B)**. An ideal consecutively double promoter expression system that can initiate the transcription of each gene under different treatments, **(C)**. An ideal simultaneous double promoter expression system that simultaneously initiates the transcription of two genes under the same condition.

Although varieties of powerfully inducible and constitutive promoters that strengthen the transcriptional intensity have been used for the production of heterologous proteins and r-proteins, developing a double-promoter expression system (DPES) with higher efficiency is still desirable (Öztürk et al., 2017). DPESs that control the transcription of the same gene through the combinatorial use of two promoters tend to result in higher extracellular protein production and more stable co-transfection. DPESs can also be used for heterologous protein expression by both prokaryotes and eukaryotes via ligation-independently cloning of the foreign gene into the dual expression vectors; this process markedly simplifies the cloning procedures, functionality expression, and protein purification process (Sinah et al., 2012). Except for typically streamlined DPESs, bidirectional promoter expression vectors featuring bidirectional initiation of the transcription of each GOI by collectively using identically constructed transcription regulator modules represent competent or higher r-protein production (Figure 1A) (Öztürk et al., 2017). For instance, a bidirectional vector containing dual cytomegalovirus promoters for the individual initiation of the expression of each GOI in a single plasmid was developed to produce recombinant antibodies; this enabled the simultaneous expression of the heavy chain and light chain for monoclonal antibody production; further, it represents competent antibody production, superior functionalities, as well as simplified manipulations compared to traditional two-plasmid co-transfection (Carrara et al., 2021). Promoter trap systems have been used to screen active promoters by cloning random DNA fragments into the multiple cloning sites of a marker gene upstream. This technique is utilized to identify active promoter elements and simultaneously obtain powerful promoters by shuffling and recombining cloned unidentified DNA fragments (Yang et al., 2013). A double or multiple promoter expression system that strongly initiates the transcription of the same target gene by using two or more promoters is expected to achieve a remarkable enhancement of heterologous protein production. Regarding different operational mechanisms, double promoter expression systems that improve foreign gene expression by elevating the transcription intensity can mainly be classified into consecutively and simultaneously operating double promoter expression systems (Öztürk et al., 2017). Consecutively operating double promoter expression systems have been generally used to prolong the transcription length through the differently phased activation of each promoter under different conditions (Figure 1B); the simultaneously operating double expression systems efficiently enhance the transcriptional strength by activating both promoters under the same circumstances (Figure 1C) (Öztürk et al., 2017).

In addition to the construction of a common bidirectional or double promoter expression vector, a promising approach to overexpress heterologous and r-proteins is the use of a synthetic promoter. Synthetic promoters can be defined by inserting functional cis-elements into the distal promoter region to either positively or negatively recruit the corresponding transcription factors, specific regulation of transcription efficiency and strength can be achieved without being constrained by endogenous factors (Machens et al., 2017; Yasmeen et al., 2023). For example, the *α*-amylase titer increased by 95% in *S. cerevisiae* by using an unfolded protein response synthetic responsive promoter, which is constructed by inserting the unfolded protein response element in front of the upstream enhancer region of yeast native promoters (Xiao et al., 2023). Artificially synthesized promoters commonly feature shorter sequences, which are more accurately regulated and more stably condition-induced for heterologous metabolic transcription in dynamic metabolic networks (Wichmann et al., 2023).

### 2.2 Fusion partners

In addition to constructing a powerful promoter for the stable and accurate transcription of foreign genes, a highly specific signal peptide (SP) or translational fusion partner (TFP) always results in more efficient secretion and higher production of the target proteins by *S. cerevisiae*. These fusion partners that are usually fused to the N-terminus of target amino acid sequences tend to be easily removed by artificially inserting a related cleavage site or a short peptide linker.

#### 2.2.1 Secretion signal peptides

An effective SP serves as the leader in orienting the target protein into the ER secretory pathway that is commonly attached to the N-terminus of the secretory protein (Bae et al., 2022). Furthermore, the efficiency and capability of translocation that greatly impacts the secretion of target proteins in both prokaryotes and eukaryotes mainly depend on the selection of SPs and secretion mechanisms (Owji et al., 2018). In addition, only if a specific SP is conferred to the target protein (von Heijne, 1990), the newly synthesized polypeptide can enter the ER secretory pathway for subsequent processes. Despite the low specificity of endogenous SPs in yeasts, the use of foreign SPs usually obtains a poorer secretion efficiency than that of SPs originating from yeast (Kaiser et al., 1987). Moreover, whether foreign signals will function in yeast remains unknown and unpredictable, and secreted signal sequences that are homologous to *S. cerevisiae* (e.g., those from acid phosphatase, *α*-factor, and invertase) are extensively used in heterologous protein secretion. Among them, the most widely used signal sequence originating from the pre-pro region of mating factor *α* (MFα) has been demonstrated to be generally applicable and functional in the expression of various heterologous proteins by *S. cerevisiae* (Ernst, 1986).

Unfortunately, the construction of a universal SP for all heterologous protein production seems to be impossible. Although a foreign SP can be expressed in a host strain (Kober et al., 2013), compared to the endogenous SP, target protein secretion is always inefficient, and the SP cannot be recognized by native translocons and cleaved off. For instance, the use of *E. coli* OmpA SP for heterologous protein production represents both a higher expression level and more efficient secretion of *Bacillus subtilis* chitosanase than *Bacillus* SP by the *E. coli* expression system (Pechsrichuang et al., 2016). Appropriate selection of SPs for heterologous proteins is often conducive to maintaining a balance between translocating efficiency and ER folding capability (Owji et al., 2018) rather than triggering a series of degradation mechanisms that lead to holistic proteolysis of both properly folded proteins and aberrantly aggregative proteins.

#### 2.2.2 Translational fusion partners

Foreign gene expression mainly hinges on the folding rate of the ER, while the secretion of heterologous proteins that are overexpressed via the ER secretory pathway always reaches saturation regardless of the posttranscriptional mechanism of *S. cerevisiae* (Schröder, 2008; Young and Robinson, 2014). Rational selection of promoters and designated modification of secretion signals are capable of enhancing heterologous protein production to a certain extent (Rakestraw et al., 2009); however, these approaches are generally not applicable to all heterologous proteins. Most studies mainly concentrated on host strain engineering, including overexpression of target genes, modulation of factors related to protein secretion, optimization of expression vector systems, and perfection of the fermentation process (Idiris et al., 2010). Compared to host strain engineering, translational fusion (in which the target gene is fused with a specific TFP) can improve target gene expression without impairing the structure of the target protein or imposing an additional burden on the ER (Bae et al., 2015).

Translational fusion is a promising expression system for enhancing the expression of both heterologous and r-proteins, in addition to using the designed fusion partners. The commonly utilized fusion system, which is a fusion tag called small ubiquitin-like modifier (SUMO), is the *S. cerevisiae* SUMO protein Smt3 and SUMO protease 1. Analogous to the removal of TFPs, the fusion tag SUMO can be removed by digestion of a highly specific SUMO protease (Lau et al., 2018), e.g., Smt3 can be deconjugated from the target protein by SUMO protease 1 (Mossessova and Lima, 2000). Similar to the functions of translational fusion, SUMO fusion is responsible for facilitating expression, increasing solubility, and simplifying purification procedures of target proteins (Malakhov et al., 2004; Marblestone et al., 2006). A stably constructed and highly expressed functional TFP that involves heterologous metabolic pathways and biosynthesis of a target protein always exhibits a remarkable upregulation of target gene expression.

#### 2.2.3 Fusion partners from highly expressed proteins

The pre-pro peptide of *S. cerevisiae* MFα has been identified as a powerful fusion partner for the production of various heterologous proteins, yet MFα is not an all-purpose secretion leader for all target proteins. Therefore, the optimal selection of a fusion partner and its appropriate modifications are deemed to directly impact secretion efficiency. The fusion partners function well for enhancing heterologous protein production, which can be mainly attributed to the increase of solubility of fusion proteins in the ER and the facilitation of transport of fusion proteins to the Golgi apparatus (Dälken et al., 2010; Xu et al., 2018). As Kex2p is a Ca^2+^-dependent serine protease that specifically recognizes dibasic residues (Lys-Arg, Arg-Arg) (Fuller et al., 1989), fusion partners can be removed by cleavage after dibasic residues in the late Golgi network through the artificial introduction of the Kex2p cleavage site. The efficient endoprotease Kex2p that was discovered in *S. cerevisiae* is a processing protease that is responsible for the proteolytic maturation of killer toxins and MFα (Kim et al., 2022). Kex2p has been used for the production of r-proteins; for example, when expressed with the MFα signal peptide that contains a Kex2p cleavage site, overexpression of KEX2 has been shown to enhance the production of the r-protein in *Pichia pastoris* (Sreenivas et al., 2015). Even though the fusion system retains the intact construction of the target protein, no fusion partner is omnipotent for all protein varieties. Fortunately, the origination of fusion partners can be generated from open reading frames that encode proteins carrying secretion signals. In addition, fusion partners can be generated from different-sized fragments of a single open reading frame, and the secretion efficiencies of different translational fusion systems always depend on the secretory capabilities of target proteins. Powerful fusion partners have been generated and screened from the known open reading frames encoding a segment of signal peptide or fusion peptides that highly secreted proteins in yeast (Bae et al., 2015).

### 2.3 Modulation of the ER secretory pathway

#### 2.3.1 Importance of the ER secretory pathway

The ER is one of the largest membrane organelles responsible for the accurate folding, posttranslational modification, and final sorting of various proteins. Newly-synthesized peptides must be properly folded before being assembled into their target organelles (Gardner et al., 2013). When secretory demands are high, protein synthesis errors may occur, resulting in the accumulation of aberrantly folded proteins. Consequently, abnormal accumulation in the ER induces an unfolded protein response (UPR), which is an adaptive signaling pathway to combat both misfolded and unfolded proteins by elevating the expression of genes that enhance the ability and efficiency of ER protein folding (Krishnan and Askew, 2014). Inevitably, a vast fraction of polypeptides will still fail to complete the appropriate conformation even under the impact of UPR (Hartl and Hayer-Hartl, 2009). Ultimately they are eliminated by ER-associated degradation, which is a signaling pathway that retro-translocates misfolded proteins back into the cytosol and then degrades them via the ubiquitin-proteasome system (Smith et al., 2011; Ruggiano et al., 2014). Additionally, unfolded proteins that cannot be transported from the ER to the Golgi apparatus accumulate in the lumen of the ER, which represents one of the rate-limiting steps in protein secretion of eukaryotic microorganisms (Lodish et al., 1983; Shuster, 1991). The ER lumen contains amounts of ER-resident protein folding chaperones and foldases that play a role in sustaining ER homeostasis by correcting both unfolded and misfolded peptides. Thus, the increase in the expression of these protein-folding chaperones and foldases is bound to exert a positive impact on the folding efficiency of heterologous proteins expressed by *S. cerevisiae*.

#### 2.3.2 ER-resident chaperone bip

The karyogamy gene Kar2, which encodes the *S. cerevisiae* chaperone binding immunoglobulin protein (Bip), is homologous to mammalian BiP and shows the highest match to it. Kar2 includes a functional secretory signal sequence that facilitates ER translocation at the N-terminus, lacks N-linked glycosylation sites, and contains an “ER retention” signal HDEL (His-Asp-Glu-Leu)/(-GCTTGACGAACT-) at the C-terminus (Normington et al., 1989; Rose et al., 1989; Kasuya et al., 1999). *Saccharomyces cerevisiae* Bip (Kar2) is the sole member of the heat shock 70 kDa protein family that is segregated into the ER for protein folding (Plemper et al., 1997; Brodsky et al., 1999). Kar2 also functions in translocating newly synthesized polypeptides across the ER membrane and recycling aberrantly folded proteins back into the ER for degradation (Normington et al., 1989; Simons et al., 1995). Furthermore, Bip transiently interacts with a variety of newly synthesized extracellular proteins but interacts more permanently with both aberrantly folded and unassembled proteins (Gething and Sambrook, 1992). Regardless of whether it is glycosylated or nonglycosylated processed, BiP binds to these aberrant proteins in the form of a stable complex (Pobre et al., 2019), leading to a reduction of the concentration of BiP in the ER lumen.

The decrease in the concentration of free BiP in the ER as well as the interaction between BiP and these aberrant proteins are intimately linked to UPR initiation. As Bip can be induced by the UPR to increase the protein folding capability of the ER, thus alleviating the high secretory demand the ER encounters; the resulting increase of Bip expression is expected to elevate the production of heterologous proteins. Bip has been demonstrated to play a positive role in heterologous protein production and the secretion of heterologous protein is proportional to the expression level of Bip (Tang et al., 2015; Zinkevičiūtė et al., 2015; Jiao et al., 2018; Jiang et al., 2023). However, overexpression of Bip in the *S. cerevisiae* expression system is not always adaptable for the secretion of heterologous protein, even negatively impairing the r-protein production. For example, no or neglectable increase was observed in the secretion of several different r-proteins with overexpression of chaperone Bip in *S. cerevisiae* (Robinson et al., 1996; Kauffman et al., 2002; van der Heide et al., 2002). A possible explanation is that Bip might more frequently bind to the unoptimized heterologous proteins, directing expressed proteins to degradation rather than to the secretory pathway (Gasser et al., 2008).

#### 2.3.3 Inducing UPR to buffer ER stress

When the ER encounters a high secretory demand or aggregation of newly synthesized polypeptides, the UPR will be triggered to buffer the folding intensity, featuring an increase in the folding capability of the ER (Menzel et al., 1997). The constitutive induction of UPR aids protein folding in the ER not only for native protein production but also for the production of heterologous proteins, which is mainly associated with the upregulated expression of foldases and ER-resident chaperones in the ER. Moreover, the disruption of the gene HAC1, which encodes the UPR regulator that upregulates the expression of folding factors in the ER, leads to a remarkable decrease in heterologous protein production (Valkonen et al., 2003).

**FIGURE 2:**
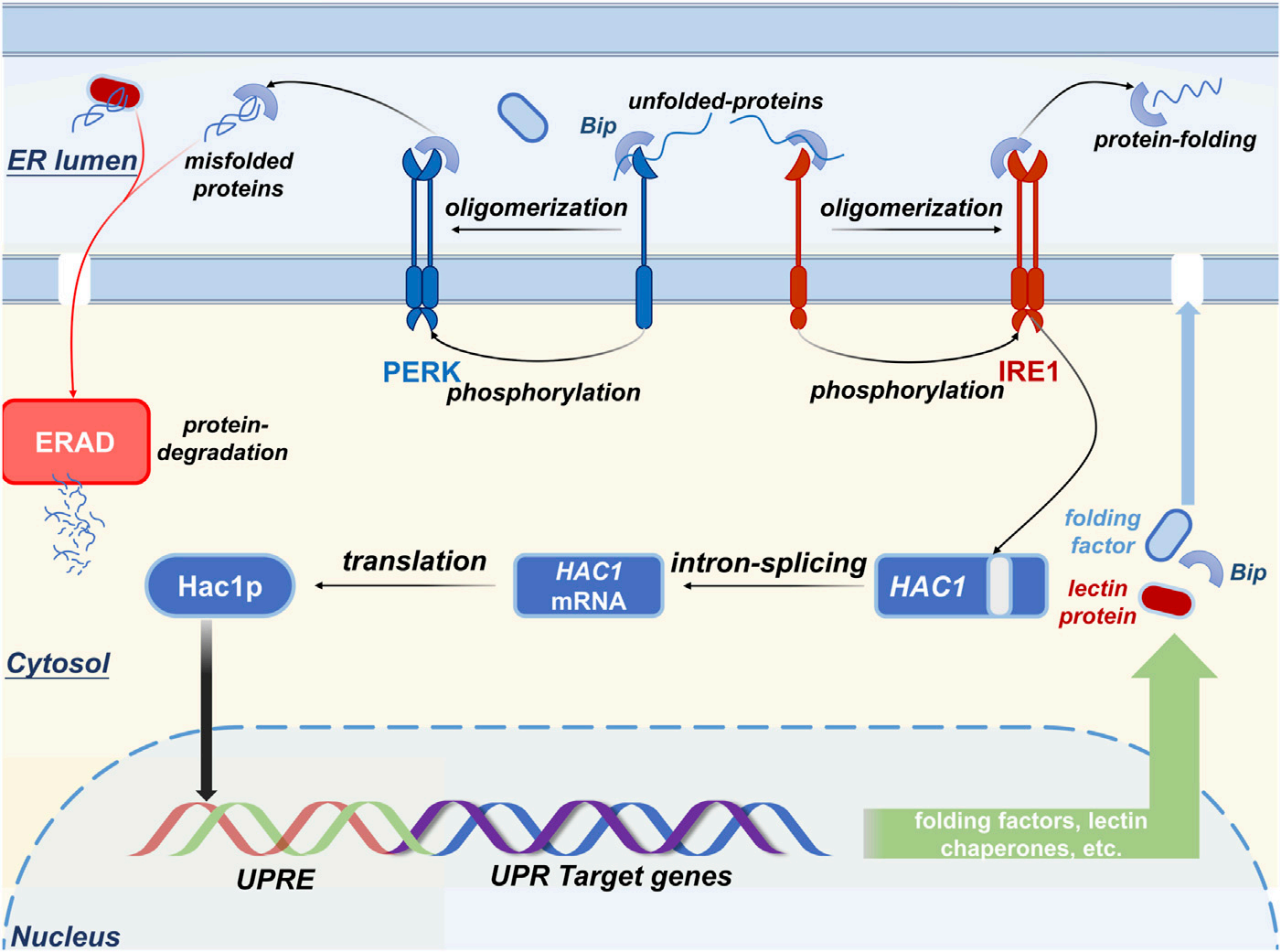
The mechanism by which IRE1, PERK, and BIP combat secretory demand. The stress sensor IRE1 is activated when the aggregation of unfolded peptides occurs in the ER lumen, leading to the initiation of the unfolded protein response (UPR) to aid the folding of the nascent peptides and ERAD to degrade the malfolded proteins. Once the UPR is activated, the expression level of the UPR target genes (e.g., resident chaperone Bip and lectin proteins, folding factors, etc.) is upregulated to buffer the ER stress.

The type-Ⅰ transmembrane protein kinase IRE1, which is also called endoplasmic reticulum to nucleus-1 protein, resides in the ER lumen where it acts as a stress sensor that transmits stress signals to the luminal domain of IRE1 in response to protein folding fluctuation (Kaufman, 1999; Patil and Walter, 2001). Another type-Ⅰ transmembrane protein kinase (PER-like ER kinase, PERK) can also be activated to combat environmental or native stresses by phosphorylating the *α*-subunit of eukaryotic translation initiation factor 2 (Shi et al., 1998), which directly inhibits the synthesis of nascent proteins (Harding et al., 1999). When secretory stress occurs (Figure 2), i) unfolded proteins trigger oligomerization and trans-autophosphorylation of both IRE1 and PER-like ER kinase and activate their RNase domains (Rubio et al., 2011; Gardner et al., 2013; Kopp et al., 2019); ii) Bip commonly binds to the luminal domain of IRE1 and under unstressed conditions, PER-like ER kinase is released to assist protein folding or to degrade aberrant polypeptides (Bertolotti et al., 2000); iii) UPR causes the reduction of free Bip in ER lumen. Activated IRE1 is responsible for the cleavage of unconventional introns that are absent from the HAC1 mRNA to maintain ER homeostasis (Sidrauski and Walter, 1997); it also encodes a functional transcription factor that belongs to the basic leucine zipper family (Nikawa et al., 1996; Sidrauski et al., 1996). This unconventional mRNA splicing increased the expression of the transcription factor HAC1 that induces UPR target genes to combat secretory demand by increasing the folding capability of ER and the expression of both folding factors and ER-resident chaperones (Kimata and Kohno, 2011; Walter and Ron, 2011).

However, forced induction or overexpression of UPR can only increase a fraction of heterologous protein production (Valkonen et al., 2003); thus, overexpression of foldases or ER-resident chaperones is not omnipotent for all foreign gene expressions. Once the translational level of a foreign gene reaches that of endogenous expression, proper folding for nascent proteins in the ER probably becomes one of the main limitations of the production of heterologous proteins. Because the efficiency of protein folding and assembly is regarded as the most critical process that is directly related to the final secretion level, modulation of the UPR pathway is expected to overcome the secretory bottleneck of heterologous proteins.

## 3 Recent technologies and future trends

### 3.1 CRISPR gene editing systems

Prokaryotic immune CRISPR/Cas systems operate as defense machinery for bacteria and archaea that degrade foreign RNA or DNA. Because CRISPR/Cas systems can recognize invading nucleic acids and degrade them (Fonfara et al., 2016), utilizing the CRISPR/Cas system for the precise cleavage of DNA at the target site vastly promotes gene editing techniques. Depending on the Cas gene sequence, repeats, and the architecture of Cas loci within CRISPR/Cas arrays (Makarova et al., 2011), CRISPR/Cas systems are classified into type I, type II, and type III, associated with the Cas3, Cas9, and Cas10 proteins, respectively. In addition, with the extensive utilization of synthetic biology, recombinant DNA technology, and precise genome editing technology such as CRISPR/Cas systems for metabolic engineering (Ullah et al., 2021; Wang et al., 2023), *S. cerevisiae* can be expected to become a promising all-producing cell factory when its metabolism is specifically redesigned.

#### 3.1.1 CRISPR/Cas9

**FIGURE 3:**
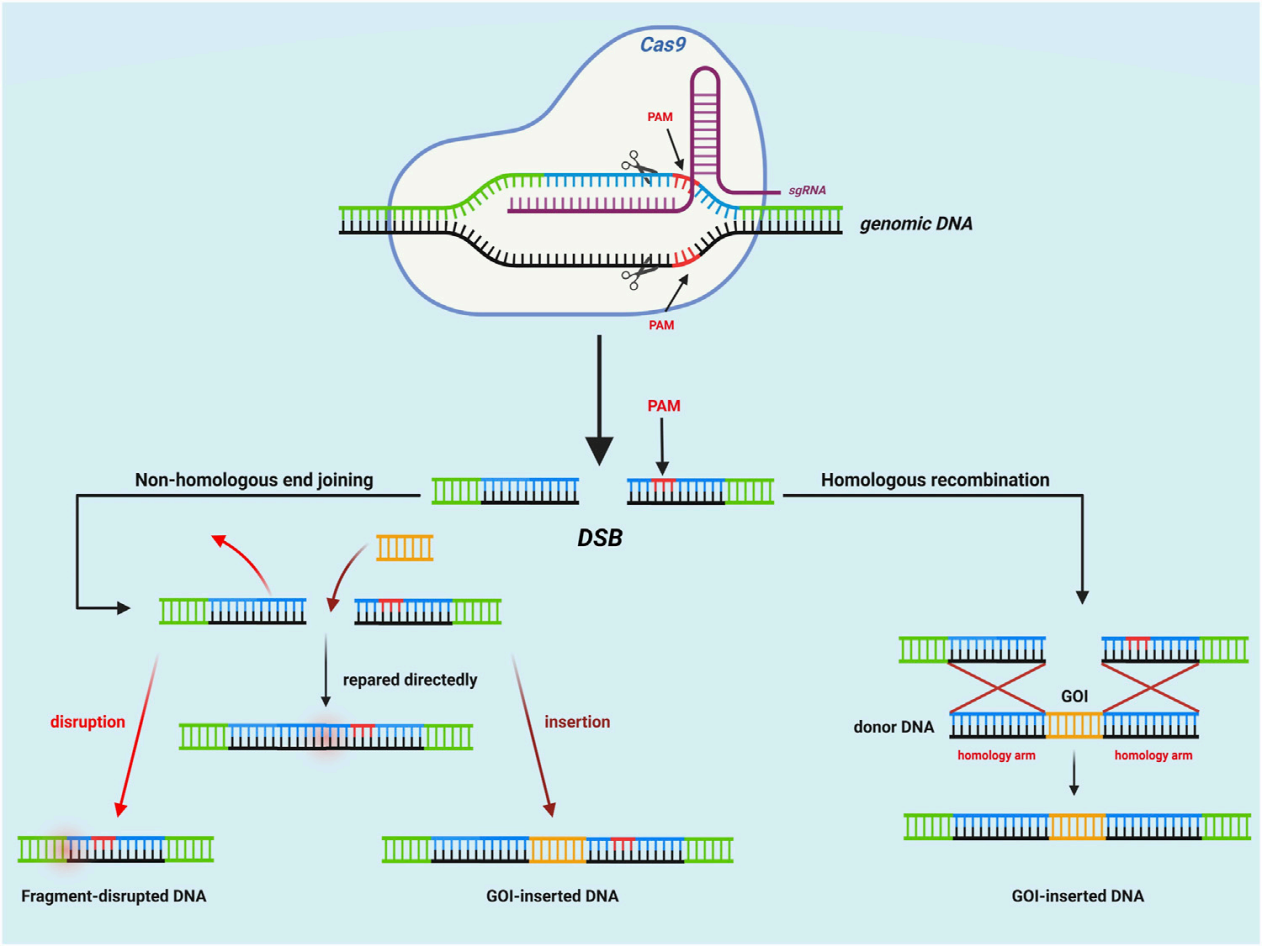
Mechanism of CRISPR/Cas9 in *Saccharomyces cerevisiae*.

Attributed to the simplicity of type II precise genome editing technology CRISPR/Cas systems—in which the CRISPR RNA-effector (crRNA-effector) functions by a single protein Cas9, which differs from that of type I and type III(Makarova et al., 2015; Rainha et al., 2020)—CRISPR/Cas9 systems have been extensively used for single- or multiple-gene disruptions, single nucleotide mutations, and gene targeting insertions. For example, the production of *α*-amylase is increased by nearly 1.8-fold in *S. cerevisiae* by CRISPR/Cas9 mediated multiplex point mutation and gene deletion (Wang et al., 2022). Also, CRISPR/Cas9-mediated heterologous expression of enzymes for metabolic regulation is widely used to enhance the production of chemicals, natural products, and therapeutics. For instance, the secretion of taxadiene synthase is improved by using an optimized genetic editing toolkit, which leads to a 25-fold increase in anti-tumor chemical taxadiene production in *S. cerevisiae* (Reider Apel et al., 2017). The CRISPR/Cas9 system that has been most widely used for heterologous gene editing is composed of: i) the Cas9 protein that is oriented to the target DNA site for the recognition of the given target DNA sequence, contains a protospacer-adjacent motif (PAM) sequence, and achieves cleavage of the target genomic DNA site (Rainha et al., 2020); ii) CRISPR RNA (crRNA) that is transcribed from the CRISPR sequence as precrRNA; iii) trans-activating crRNA (tracrRNA) for the maturation of crRNA via the activation of the conserved RNase III(Deltcheva et al., 2011); iv) a donor DNA template containing homology arms in 5′ and 3’ as well as carrying a GOI. Briefly, the type II genome editing technology functions in the direction of the Cas protein to interact with tracrRNA-crRNA duplexes; then, a double-stranded break (DSB) is created that is repaired via nonhomologous end-joining (NHEJ) and/or homologous recombination (HR) at the target gene site for the precise deletion or insertion of genes (Figure 3).

The most extensive utilization of a single guide RNA (sgRNA) in *S. cerevisiae* increases the efficiency of gene editing by the CRISPR/Cas9 system. For example, *Ryan* et al. developed multiplex CRISPR, utilizing a self-cleaving hepatitis delta virus ribozyme that is fused to sgRNA for the removal of redundant RNA sequence, achieves nearly 100% efficiency of the CRISPR/Cas9 mediated homologous direct targeting by a single sgRNA (Ryan et al., 2014). The guide RNA that is composed of a scaffolding RNA (which is required for forming the Cas9/gRNA complex) and a 20 bp gRNA spacer sequence (which is complementary to the target DNA site that directs the Cas protein to the target DNA PAM site) consolidates the functions of both crRNA and tracrRNA. Additionally, the induction of DSBs, which is generally regarded to cause genome lesions that threaten genome stability and cell survival (Gnügge and Symington, 2020), is also applicable for genomic integration resulting from the highly efficient HR repair of *S. cerevisiae*; this repair maintains high integrity of the host genome and achieves high-fidelity repair of DSBs by using donor DNA (Lisby et al., 2001; Wright et al., 2018). Because of the specific PAM sequence that is recognized by the Cas protein, the efficiency and accuracy of both-end design for gRNA always constrain the genome editing capability and efficiency when using the CRISPR/Cas9 system, especially for multiplex genome editing (Adiego-Pérez et al., 2019). Therefore, computer-aided design or other auxiliary tools for the desired gRNA that are appropriate for a variety of Cas proteins are required for efficient genome editing and multiplex gene integration (Bourgeois et al., 2018; Li et al., 2020). For example, the programmable red light switch PhiReX 2.0 has been developed to rewire metabolic fluxes by targeting endogenous promoter sequences through sgRNAs, which leads to 6-fold upregulation of a native promoter gene (Machens et al., 2023). 

#### 3.1.2 CRISPR/Cpf1 system

Biosynthetic techniques based on the CRISPR/Cas9 system are expected to achieve multiplex genetic manipulations and genome integration without the need for additional repeated gene edits and selective markers. The single RNA-guided Cpf1 endonuclease that was defined as a class 2/type V CRISPR/Cas system shows specificity to several bacteria and an exceptional archaeon (Candidatus Methanomethylophilus alvus) (Vestergaard et al., 2014; Makarova et al., 2015); it was classified as the effector protein Cfp1 that is responsible for target cleavage of the PAM sequence and crRNA processing (Makarova et al., 2017). The following presents developments and features that differ from those of the type II CRISPR/Cas system: i) Cpf1 protein is guided only by crRNA without the requirement for a tracrRNA compared to a gRNA that is required for Cas9(Zetsche et al., 2015); ii) the T-rich PAM sequence of the Cpf1 protein is located at the 5′ end of the target DNA, which contrasts with the location of Cas protein at the 3’ end of target DNA (Verwaal et al., 2018); iii) Cpf1 protein is responsible for processing pre-crRNA to the mature state and can be expected to simplify the multiplex genome editing procedures as well as facilitate the development of the CRISPR/Cas gene editing system.

Based on a self-cloning CRISPR/Cas9 system (scCRISPR/Cas9), which contains a self-cleaving palindromic sgRNA plasmid that is repaired by HR for CRISPR/Cas9-mediated genomic mutation and site-specific knock-in transgene creation (Arbab et al., 2015), the scCRISPR/Cpf1 system facilitates the genome editing process by circumventing any cloning step; this circumvention leads to highly efficient singleplex and tripleplex genomic integration (Li et al., 2018).

#### 3.1.3 Multiplex genomic editing system

**FIGURE 4:**
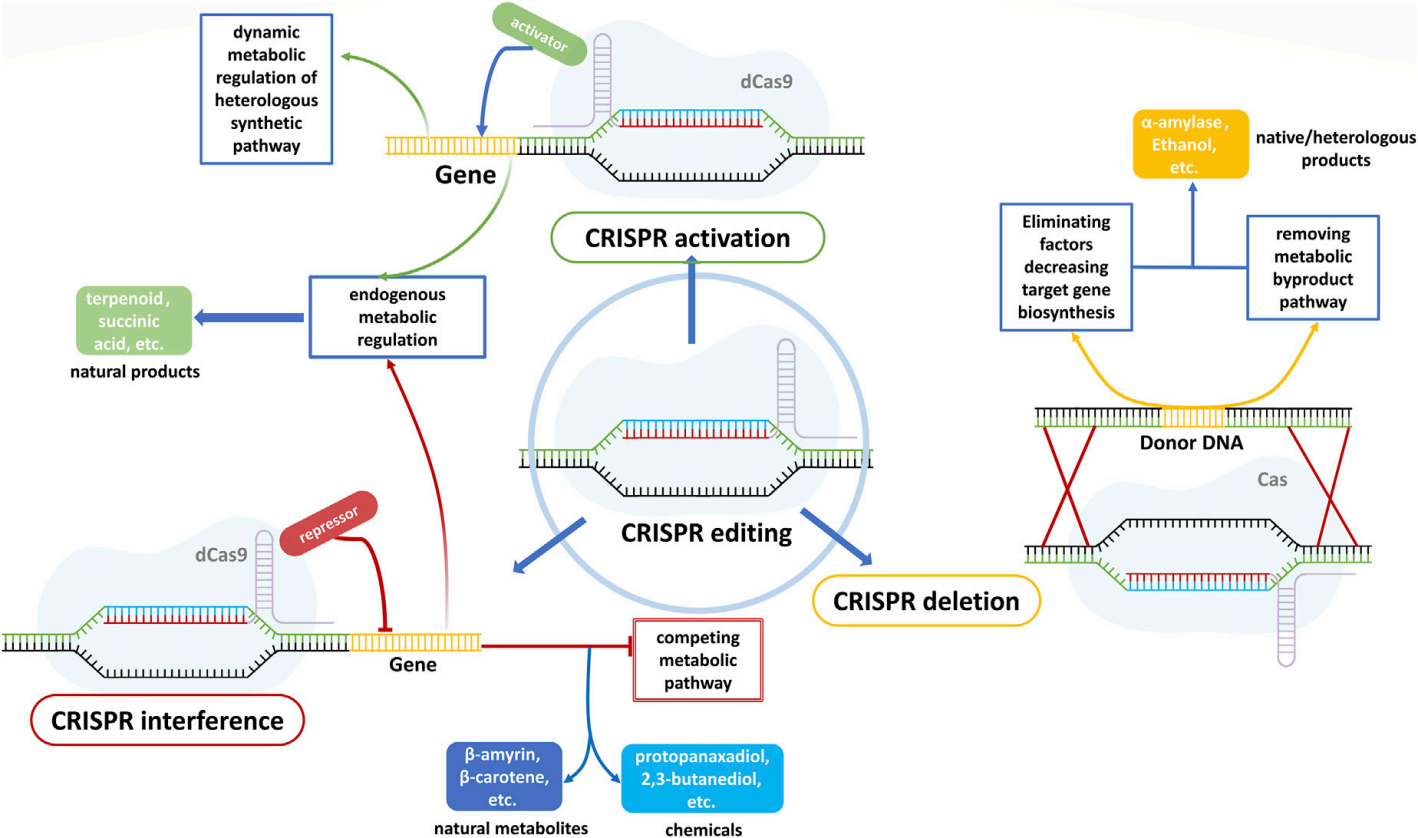
The mechanisms and applications of CRISPR activation(a), interference (i), and deletion (d). CRISPRa is expected to enhance both native and foreign gene expression by fusing an activation domain to the nuclease-deficient Cas protein (Shaw et al., 2022; Sugianto et al., 2023). By fusing a repression domain to the nuclease-deficient Cas protein, CRISPRi is applied to inhibit the metabolic competing pathways, leading to an increase in the biosynthesis of natural metabolites and chemicals (Ni et al., 2019; Lim et al., 2021; Morita et al., 2022). Overexpression of target genes is always not allowed in most microorganisms due to the endogenous defensive mechanism and complex metabolic networks, thus using CRISPRd to remove the competing pathways and factors limiting protein secretion always can increase target gene production (Wang et al., 2022; Yang et al., 2022).

Microorganisms have evolved to combat environmental stresses with the goal to maintain metabolic homeostasis, which hinders the realization of high titer, rate, and yield for target proteins, and *S. cerevisiae* is no exception. Hence, rewiring the metabolism of *S. cerevisiae* is needed to extend substrate ranges, improve cellular properties, obtain new capabilities, enhance endogenous or heterologous protein production, and tolerate internal or external stresses (Du et al., 2011; Hong and Nielsen, 2012; Jens and Keasling, 2016; Lian et al., 2018). Genomic editing technologies are derived from CRISPR/Cas9 systems, including multifunctional CRISPR systems with truncated gRNAs without creating DSBs(Figure 4): i) tri-functional CRISPR-AID combines transcriptional activation (CRISPRa), transcriptional interference (CRISPRi), and gene deletion (CRISPRd). It achieves the phenotype desired by combinatorial metabolic engineering by generating possible gRNA combinations in a single system that uses three orthogonal nuclease-deficient CRISPR proteins to prevent crosstalk between gRNAs(Lian et al., 2017; Schultz et al., 2018); ii) tri-functional CRISPR-ARE combining CRISPRa, transcriptional repression (CRISPRr), and genome editing (CRISPRe) achieve the combinatorial metabolic engineering of non-model organisms by using a single nuclease-active Cas9-VPR fusion protein (compared to three independent PAM-recognizing CRISPR proteins of CRISPR-AID) (Dong et al., 2020). Moreover, EvolvR, which combines the target specificity of CRISPR/Cas systems, enables random or target insertion by an error-prone DNA polymerase in a single-stranded break. This break is created with a nicking variant of Cas9 (nCas9) when native homology repair is initiated (Halperin et al., 2018). EvolvR-mediated mutagenesis is expected to realize continuous target nucleotide diversification and multiplex gene mutations in higher eukaryotes, e.g., yEvolvR applied in *S. cerevisiae* (Tou et al., 2020). Another CRISPR/Cas9-based multiplexed integration system features in recruiting Rad51 recombinase to the proximity of the DSBs by fusing a protein Brex27 to Cas protein, which enhances homologous recombination and achieves 78% quadruple integration efficiency in *S. cerevisiae* (Meng et al., 2023). 

Remarkably, because of the complicated cellular metabolism and intimate interaction between gene expression and metabolites, the changes that various modifications impose on metabolic pathways are unpredictable.

### 3.2 Fusion partners for efficient genetic editing

Although the robust homology-directed repair (HDR) capability of *S. cerevisiae* provides efficiency for accurate gene disruption, insertion, and mutation, multiple gene edits usually require a more powerful HR procedure because of the low efficiency of gene targeting. Further, DSBs generated by precise splicing of Cas9 can also be repaired by NHEJ as it initiates directed ligation of broken ends without the need for donor DNA templates. Both the initial processing of DSB ends (including the resection of DSB ends and the formation of 3′ short overhangs at the end-resected DSBs) are required to initiate HDR. Therefore, accelerating the generation of 3′ short overhangs as the DSBs generated by Cas9 splicing can be expected to enhance HR while repressing NHEJ (Zhang et al., 2022). The conserved complex Mre11-Rad50-Xrs2 of *S. cerevisiae* is responsible for the recognition of the DSBs and is recruited to the broken ends for the initiation of DSBs resection. This recruitment leads to the repair of DSBs via HR, which repairs the obliterated DNA information by utilizing the stored genetic information of the homologous double-strand DNA (Casari et al., 2019). Furthermore, the endogenous exonuclease Mre11 of the complex Mre11-Rad50-Xrs2 enables the initiation of the cutting of DSB ends because of its single-strand DNA endonuclease activity and 3′-to-5′ exonuclease activity (Casari et al., 2019; Zhang et al., 2022). To achieve more efficient, accurate, and stable genome multi-editing procedures for metabolic regulation, the fusion of endogenous exonuclease or HR factors and Cas9 facilitates the HDR procedure.

#### 3.2.1 Nuclease-deficient CRISPR protein fusion strategies

Numerous specifically developed promoter expression systems and pathway modification procedures have been used to enhance the gene transcription level for metabolic engineering; however, the transcriptional regulation efficiency for the complex metabolic pathway remains relatively low. The direct fusion of functional units (activator or repressor) and nuclease-deficient CRISPR proteins that have been used in CRISPRa/i exhibit highly precise and efficient transcriptional regulation. For example, the fusion of location functional units that can orient metabolites to target metabolic pathways to the deactivated Cas9 (dCas9) is expected to reduce the residence of bioproducts and facilitate the elucidation of unclear mechanisms (Xie et al., 2020), leading to efficient secretion and discovery. However, single-effector fusion and finite fusion partners are always unsuitable and incompetent for the efficient regulation of complicated metabolic pathways and multi-gene regulation. A CRISPR-mediated regulator recruiting system has been developed to amplify transcriptional activation and repression. This system has been developed by fusing protein scaffolds (SPY and Sun Tag systems) with nuclease-deficient CRISPR proteins so that several effectors are recruited for accurate and efficient multi-regulation of target gene transcription (Zhai et al., 2022).

#### 3.2.2 Nucleotide-level fusion strategies

Even though protein fusion strategies can achieve a substantial improvement of genetic manipulation efficiency, nucleotide-level fusion strategies can also achieve efficient genome modulation while leaving out the translation. The synthetic sRNA-mediated gene regulation system achieves the repression of target gene expression at the post-transcriptional level with the regulator Hfq protein (Yeom et al., 2022), which involves the binding of sRNA and target mRNA. Moreover, the synthetic sRNA assembled with a specifically modified mRNA-binding module sequence can be expected to bind to any target mRNA via base-paring, resulting in specific repression and precise regulation of metabolic pathways. To simultaneously regulate multiple target genes, the cloning of several synthetic sRNAs to a vector or the use of several sRNA vectors are conventional processes. However, the repeated use of the same scaffold sequences always leads to low regulatory efficiency, which can be attributed to unintentional HR. Therefore, the fusing of several mRNA binding modules that are specific for target genes to a single sRNA scaffold can achieve multiplex gene regulation, thus simplifying genetic procedures while imposing no additional burden on the cell (Yeom et al., 2022).

### 3.3 Codon optimization

Heterologous metabolic regulation has been crucial in *S. cerevisiae* expression systems, and regulation effectors are generally proteins. In the translation process, 64 codons that encode 20 amino acids, three stop codons, and transfer RNAs(tRNAs) are responsible for deciphering the genetic information contained in messenger RNA (mRNA) and the transition of nucleotide triplets (codon) into a specific amino acid sequence (Pinkard et al., 2020). More frequently used genetic codons in the genome are always located in highly expressed genes, where they function in facilitating gene translation. Rare genetic codons are commonly used in genes with low expression, representing a lower translation rate and protein synthesis. Further, codon usage bias, which is regarded as the rate-limiting factor in the translation process, determines the transcription and translation efficiency, as well as the protein folding rate in the target gene expression process (Tuller et al., 2010; Xu et al., 2021; Parvathy et al., 2022). Incompatible codon usage can even impair protein synthesis and impact the conformation and functionality of expressed proteins. The same amino acid is allowed to be encoded by synonymous codons (e.g., Gly encoded by GGU, GGC, GGA, and GGG) because of the degeneration of genetic codons, which do not change the primary amino acid sequences or protein conformation. Therefore, codon optimization by synonymous mutations alters the DNA and RNA coding sequence via point mutations (usually mutations in the third base of codons to maintain genetic stability) to adapt to the tRNA abundance or codon frequencies in target species cells (Karaşan et al., 2022). This mechanism is expected to significantly promote the translation efficiency in genetic manipulations of metabolic regulation. Relative synonymous codon usage and codon adaption index have been used to measure the extent of non-random usage of synonymous codons for specific coding sequences (Sharp et al., 1986; Rong et al., 2023). This refers to the prediction of the synonymous codon usage bias of a given amino acid sequence and the evaluation of the resemblance between the synonymous codon usage bias of a target gene and the synonymous codon frequency of a highly expressed genomic gene. However, appropriately elevating relative synonymous codon usage or codon adaption index does not always lead to enhanced protein biosynthesis. Therefore, codon optimization procedures utilize codon harmonization, to match the native codon usage frequencies with those of the target expression host more closely. This strategy is particularly effective in the low translation rate of heterologously expressed proteins (Punde et al., 2019). For example, the all-purpose and powerful algorithm CHARMING has been developed to design codon-harmonized gene sequences for heterologous gene expression by substituting synonymous codons to match the codon usage bias of the target genome (Wright et al., 2022). Hence, genome-adapted codon optimization and harmonization can be expected to enhance gene expression, improve translation efficiency, reduce structure impediments of heterologous mRNA, and prevent deficient biosynthesis.

## 4 Conclusion and perspective

*Saccharomyces cerevisiae* has been extensively used for the production of numerous biologics, including natural products, therapeutic products, medicinal products, industrial products, commercial products as well as other r-proteins (Gerngross, 2004; Ferrer-Miralles et al., 2009; Krivoruchko and Nielsen, 2015; Li and Borodina, 2015). Metabolic engineering has been employed for heterologous synthesis to counter the increasing demand for therapeutics and pharmaceutical chemicals that are generated from secondary metabolites (Rahmat and Kang, 2020). Consequently, this represents a promising field for biosynthetic engineering.

The galactose-inducible promoter is a transcriptional control element that has been extensively applied in metabolic engineering and biosynthetic engineering to initiate heterologous genes in *S. cerevisiae*. This promotor has been demonstrated to be unsuitable for the repeated expression of foreign genes because of the genetic instability caused by the homologous recombination of *S. cerevisiae* (Peng et al., 2018). Thus, artificial genetic engineering (i.e., the amplification of a haplo-insufficient gene) was developed for the multi-integration of heterologous genes into the yeast genome. This amplification of a haplo-insufficient gene is attributed to its regulatable promoter strength and transcriptional efficiency, auto-selectable maintenance of heterologous copy numbers without the addition of antibiotics, and sufficient loci for gene amplification (Peng et al., 2022b). Multiplex genetic regulation and edits for more efficient and valuable bioproduction remain challenging because of the complexity of genetic and metabolic networks. The multifunctional CRISPR systems CRISPR-AID and CRISPR-ARE that contain truncated gRNA and require no DSB introduction, strongly facilitate genomic editing procedures from gene insertion to target mutation compared to the traditional gene editing technologies (Table 1). This leads to a more efficient and applicable genomic technique that expands the CRISPR library. In addition, emerging genomic technologies based on CRISPR/Cas9 systems that contain a self-splicing sgRNA or a single multifunctional gRNA contribute to the simplification of repeated genomic edits that always require several rounds of repetitive screening processes. Because of its role in the conserved post-transcription modification processes, cell surface glycosylphosphatidylinositol-anchoring technology is expected to aid the construction of novel functional yeast cells with oriented protein transportation and accurate colocalization. Their construction depends on the functional proteins that reside on the plasma membrane (Inokuma et al., 2021; Aguilera-Romero and Muñiz, 2023).

**TABLE 1:** Gene editing technologies in *S.cerevisiae*.

Number	Technology	Mechanism	Advantages	Disadvantages	Ref
1	High-fidelity homology-directed repair	homologous recombination	precision, versatility, simplicity	low efficiency, off-target effects	Yeh et al. (2019)
2	Cre/LoxP	bacteriophage P1 enzyme and Cre recombinase-mediated site-specific recombination	high-specificity, recyclable marker	time-consuming and costly, off-target effects	Carter and Delneri (2010)
3	I-SceI	site-specific DNA endonuclease-mediated DSB	precision, efficient gene targeting	limited targeting sites, off-target effects	Storici et al. (2003)
4	Transcription Activator-Like Effector Nucleases (TALENs)	TALE nuclease mediated DSB	precision, versatility, High targeting specificity	complex TALEN design, time-consuming and costly	Joung and Sander (2013)
5	Zinc-Finger Nucleases (ZFNs)	ZFNs-mediated site-specific DSB	high targeting specificity, versatility, low off-target effects	time-consuming and costly design of ZFNs	Urnov et al. (2010)
6	Base editing	DNA base direct conversion	precise single-base modification, high targeting specificity, and efficiency, no DSB	off-target effects, Low applicability	Marx (2018)
7	Homing Endonucleases/Meganuclease	occurring endonucleases-mediated DSB	high targeting specificity, low off-target effects	complex and time-consuming design	Chevalier and Stoddard, (2001)
8	FLP/FRT	FLP recombinase-mediated site-specific recombination	precision, high specificity, and efficiency	low applicability	Park et al. (2011)
9	CRISPR/Cas	gRNA mediated DSB	high precision, high targeting specificity, and efficiency, multiplex editing, economy	off-target effects, requiring unique gRNA design	Verwaal et al. (2018); Rainha et al. (2020)

Newly developed technologies, including DPESs, fusion strategies, fast and simple genome editing, metabolic engineering, and codon optimization can be expected to facilitate genetic manipulation procedures and enrich the gene editing library not only for yeast, but also for other fungi. For example, DPESs achieve precise up-and down-regulation of heterologous metabolic synthetic pathways in *B. subtilis* and *E. coli*that enhancer-proteinproduction. The stability of the mRNA level, translation efficiency, and expression level of heterologous genes could be enhanced by using optimized codons in filamentous fungi (Liu et al., 2023). Also, CRISPR/Cas9 systems have been extensively used to perform efficient gene modification in filamentous fungi of the *Aspergillus* genus for the biosynthesis of natural and recombinant bioproducts (Jin et al., 2022). Further, promising fusion strategies combined with different synthetic effectors are expected to have wide application prospects in a variety of scenarios. These strategies contribute to the construction of multifunctional or specific modules for genetic manipulation and metabolic regulation, thus facilitating editing procedures and a more accurate modulation of metabolic networks.
